# Identification of senescence-related lncRNA prognostic index correlating with prognosis and radiosensitivity in prostate cancer patients

**DOI:** 10.18632/aging.204888

**Published:** 2023-09-23

**Authors:** Dechao Feng, Li Li, Xu Shi, Weizhen Zhu, Jie Wang, Ruicheng Wu, Dengxiong Li, Wuran Wei, Ping Han

**Affiliations:** 1 Department of Urology, Institute of Urology, West China Hospital, Sichuan University, Chengdu 610041, China

**Keywords:** prostate cancer, senescence-related lncRNA prognostic index, biochemical recurrence, radiosensitivity, androgen response

## Abstract

Background: An increasing number of studies are shown how crucial a role cellular senescence plays in tumor development. In this study, we developed a senescence-related lncRNA prognostic index (SRLPI) to forecast radiosensitivity and the probability of biochemical recurrence (BCR) in patients with prostate cancer (PCa).

Methods: PCa cohorts in TCGA and GEO databases were downloaded. Senescence-and prognosis-related lncRNA with differential expression in tumor and normal samples were identified and used to establish the SRLPI score. Mutation landscape, function pathway, tumor stemness and heterogeneity and tumor immune microenvironment were also analyzed. We performed the analysis using R 3.6.3 and the appropriate tools.

Results: A SRLPI score was constructed based on SNHG1 and MIAT in the TCGA cohort. Our classification of PCa patients into high- and low-risk groups was based on the median SRLPI score. When compared to the low-SRLPI group, the high-SRLPI group was more vulnerable to BCR (HR: 3.33). In terms of BCR-free survival and metastasis-free survival, the GSE116918 showed similar findings. Surprisingly, the SRLPI score demonstrated a high level of radiosensitivity for diagnosis (AUC: 0.98). Age, Gleason score, T stage, N stage, positive lymph nodes, and residual tumor were all significantly greater in patients with high SRLPI scores. Furthermore, this score was significantly related to markers of senescence. Protein secretion and androgen response were found to be substantially enriched in the low-SRLPI group, whereas E2F targets were found to be strongly enriched in the high-SRLPI group for pathway analysis. For the tumor microenvironment assessment, B cells, CD8+ T cells, immune score and TIDE score were positively related to SRLPI score while endothelial level was negatively associated with SRLPI score with statistical significance.

Conclusions: We developed a SRLPI score that was related to prognosis and radiosensitivity and might be helpful in clinical practice.

## INTRODUCTION

Population aging is steadily elevating to a significant worldwide concern. Statistics show that those over the age of 65 have an 11-fold higher incidence of cancer than people under that age [1]. Prostate cancer (PCa), which is most prevalent in males 65 and older, will exponentially increase in prevalence by 2030, when almost 20 percent of the world’s population will be 65 or older [2, 3]. In light of this, PCa remains a public health concern for men, and as the world’s population ages, its effects will become more obvious [4]. For low- and intermediate-risk localized PCa patients, radical prostatectomy and radical radiotherapy are the two chosen treatments [5–8]. Despite surgery and radiation, about 27–53% of these patients still succumb to the disease due to biomedical recurrence (BCR) or metastasis [7, 9–12]. Additionally, roughly a third of patients who experience recurrence eventually develop castration-resistant PCa, the most common kind of cancer fatality with an estimated mortality rate of nearly 28 percent for 5-year survival [6, 12]. The clinical variability of PCa is paralleled in the geographical and clonal genetic variety, making PCa a heterogeneous disease [4]. Therefore, it is crucial to include molecular biomarkers that forecast BCR in clinical therapies to stop further development or metastasis.

Stress-induced permanent, irreversible cell cycle arrest known as cellular senescence causes a reduction in cellular processes such proliferation, migration, homing, and differentiation [8, 13–17]. Senescence brings about a number of abnormalities in the human body that have been demonstrated to hasten aging, cause carcinogenesis, and promote the spread of cancer [1]. Furthermore, aging is an established risk factor for many tumors [18, 19]. Therefore, it is crucial to investigate the intersections between aging and cancer and look for new ways to heal those intersections in order to combat malignancies that are linked to aging. Long non-coding RNAs (lncRNAs), a category of non-coding RNAs longer than 200 nucleotides, can regulate the expression of protein-coding genes and have come to light in recent years as having significant involvement in a number of biological processes related to human diseases, including PCa [20–25]. In our earlier researches, we discovered certain senescence-related biomarkers linked to PCa patients’ prognosis [8, 14, 15], but the potential effects of lncRNAs associated with senescence-related genes on this disease have not been reported. Thus, in this study, we established a senescence-related lncRNA prognostic index (SRLPI) to predict BCR risk and radiosensitivity for PCa patients.

## METHODS

### Data preparation

We downloaded 279 genes responsible for cellular senescence in humans from the CellAge database (http://genomics.senescence.info/cells) which was based on gene manipulation experiments in different human cell types [26]. The PCa gene matrix and clinical characteristics from our prior work were used in The Cancer Genome Atlas (TCGA) database [10]. We examined differentially expressed lncRNAs and lncRNAs associated with BCR. Differential expression was defined as when Padj was less than 0.05 and the fold change absolute value was larger than 1.5. The senescence-related lncRNAs were calculated using a Pearson analysis, and the requirements were that the *P* value be less than 0.5 and the absolute value of the coefficient be greater than 0.4. A Pearson analysis was used to determine the senescence-related lncRNAs, and the *P* value and absolute value of the coefficient had to be less than 0.5 and more than 0.4, respectively. 430 samples from the TCGA database were examined, and the log-rank test for BCR-free survival yielded a *p* value less than 0.05. We discovered the lncRNAs that were used to build the SRLPI score after the intersection of differentially expressed BCR-related, senescence-related, and lncRNAs. We then performed lasso and multivariate Cox regression analysis. The prognostic and clinical values of SRLPI in 430 PCa patients in TCGA database were analyzed, which was confirmed by the GSE116918 [27] and GSE70768 [28]. Additionally, in order to determine the prognostic role of the SRLPI score, we graded the PCa patients in TCGA database or GSE116918 [27] based on the European Association of Urology (EAU) [29] and National Comprehensive Cancer Network (NCCN) guidelines [30] and further compared these factors using Cox regression analysis in terms of BCR-free survival. The diagnostic ability of SRLPI for radiosensitivity was analyzed by the GSE53902 [31]. Furthermore, we explored the relationship between SRLPI score and senescence markers, including p16, p21, CTSD, LMNB1 and RB1, based on the previous study [32].

### Mutation landscape and functional analysis of SRLPI

The TCGA database, which contains information on PCa, was used to download RNA-sequencing profiles, genetic mutations, and related clinical data. Using the maftools package in the R programming language, the data of mutations were downloaded and displayed. A comparison of the differences in mutation frequency between the two kinds was also done.

In terms of functional analysis, gene set variation analysis was performed using “h.all.v7.4.symbols.gmt “and “h.all.v7.4.symbols.gmt “from the molecular signatures database [33, 34]. The minimal gene set was determined to be 5 and the maximum gene set to be 5000 based on gene expression. Each sample’s enrichment score was determined, and using the Wilcox. Test function, we analyzed the difference between samples with high and low SRLPI scores as specified by the median value. Statistical significance was defined as a Padj value of less than 0.01 and an absolute fold change value greater than 1.5.

### Tumor stemness and heterogeneity analyses

Tumor stemness indexes included differentially methylated probes-based stemness scores (DMPss), DNA methylation-based stemness scores (DNAss), enhancer elements/DNA methylation-based stemness scores (ENHss), epigenetically regulated DNA methylation-based stemness scores (EREG-METHss), epigenetically regulated RNA expression-based stemness scores (EREG.EXPss) and RNA expression-based stemness scores (RNAss) [35]. Tumor heterogeneity included homologous recombination deficiency (HRD), loss of heterozygosity (LOH), neoantigen (NEO), tumor ploidy, tumor purity, mutant-allele tumor heterogeneity (MATH), tumor mutation burden (TMB) and microsatellite instability (MSI) [36, 37]. The results of above indicators were obtained from our previous study [7]. We compared the differences of high- and low- SRLPI groups using the Wilcoxon rank sum test.

### Tumor microenvironment assessment

**Figure 1:**
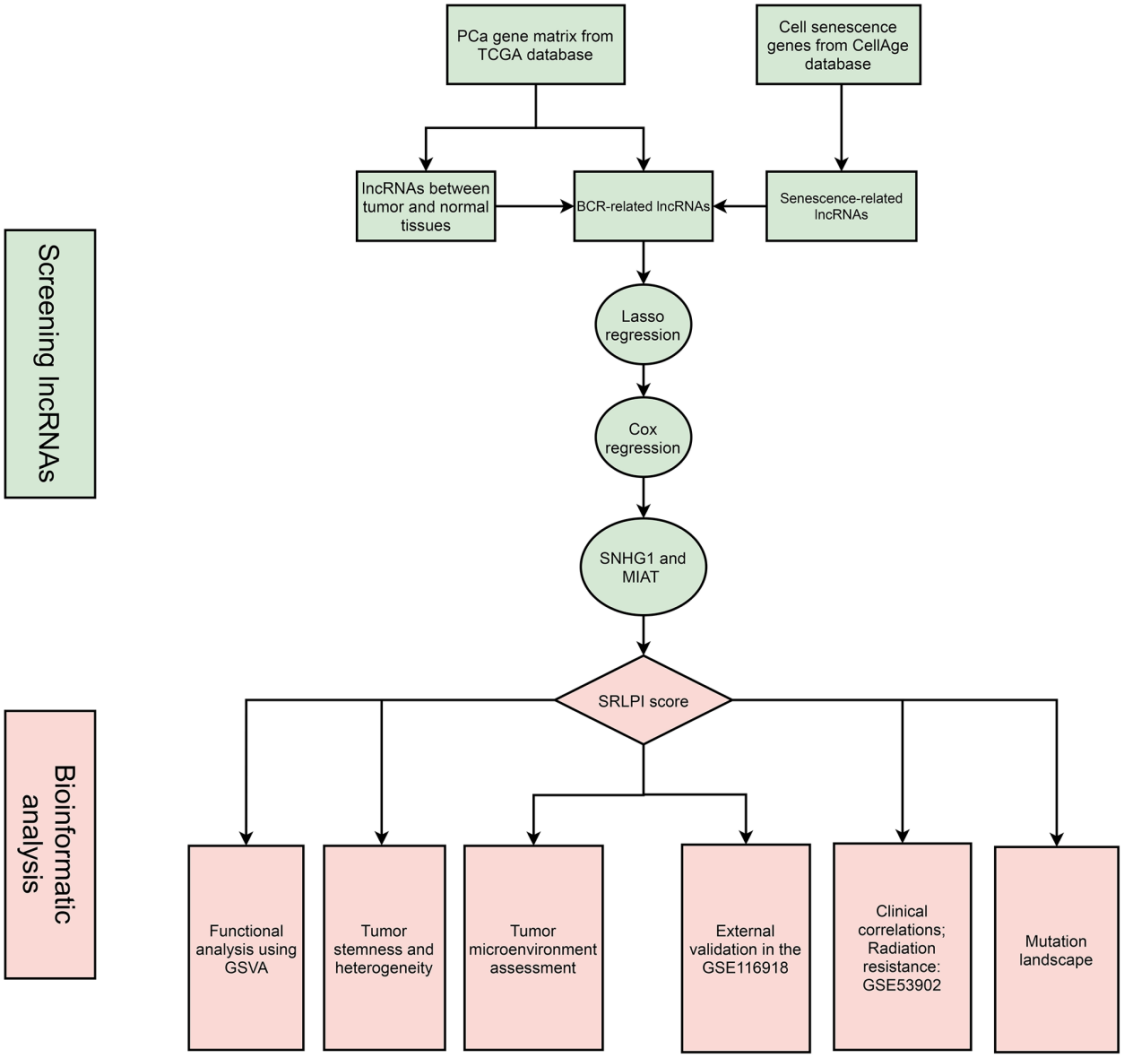
**The flowchart of this study.** Abbreviations: PCa: prostate cancer; lncRNA: long non-coding RNA; BCR: biochemical recurrence; GSVA: gene set variation analysis; SRLPI: senescence-related lncRNA prognostic index.

EPIC and ESTIMATE algorithms were used to assess the entire tumor microenvironment and immunological components [38–40]. The tumor immune dysfunction and exclusion (TIDE) algorithm was used to predict the potential response to immune checkpoint blockade (ICB) therapy [41]. A high TIDE score indicates low ICB efficacy. The Wilcoxon rank sum test was used to examine the differences in 54 immune checkpoints and tumor microenvironment scores between groups with high and low SRLPI scores. The study’s flowchart is shown in Figure 1.

### Statistical analysis

We performed the analysis using R 3.6.3 and the appropriate tools. The *t*-test was used to compare two groups where the variable was of the numerical type, and one-way ANOVA was used to compare three groups when the data passed the tests for normal distribution and homogeneity of variance. Welch *t*’ test and Welch one-way ANOVA were employed for two-group comparisons and three-group comparisons, respectively, where the data satisfied the criteria for normal distribution but failed the homogeneity of variance test. Because the normal distribution was not satisfied, Wilcoxon was used for comparisons between two groups, and Kruskal-Wallis was used for comparisons between three groups. When the data matched the criteria of theoretical frequency >5 and total sample number ≥40 and the variable was categorized, the chi-square test was employed to compare the groups. The continuous adjustment chi-square test (Yates’ correction) was employed to compare groups when the data matched the criteria of 5> theoretical frequency ≥1 and total sample number ≥40. Fisher’s exact test was employed to compare groups where the theoretical frequency was less than 1 or the total sample size was under 40. A Kaplan-Meier curve representing the results of the log-rank test was used for the survival analysis. The threshold for statistical significance was two-sided p 0.05. Significant marks were as follows: not significance (ns), *p* ≥ 0.05; ^*^p < 0.05; ^**^p < 0.01; ^***^p < 0.001.

### Availability of supporting data

The datasets presented in this study can be found in online repositories. The names of the repository/repositories and accession number(s) can be found in the article/supplementary material.

## RESULTS

### SRLPI identification and its clinical applications

**Figure 2:**
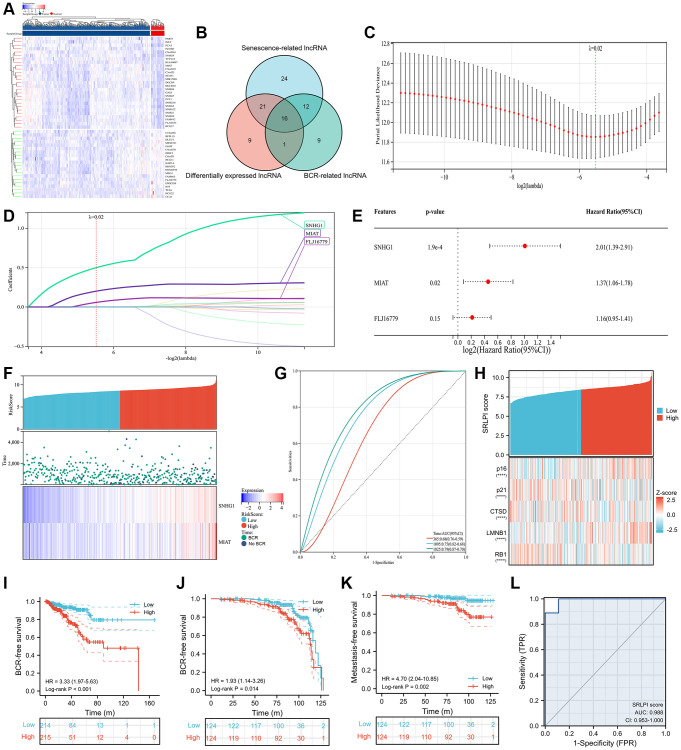
**SRLPI identification and its clinical applications.** (**A**) heatmap showing differentially expressed lncRNAs between tumor and normal samples in TCGA database; (**B**) Venn plot showing the intersection of senescence-related, differentially expressed and BCR-related lncRNAs; (**C**) lasso regression analysis showing the optimal lambda for the model; (**D**) lasso regression analysis showing the lncRNAs in the optimal model; (**E**) multivariate Cox regression analysis showing the prognostic lncRNAs used to construct the SRLPI score in terms of biochemical recurrence-free survival; (**F**) risk factor plot showing the distribution of outcomes and final lncRNAs in the SRLPI score; (**G**) time-dependent ROC curve showing the diagnostic ability of SRLPI score for BCR identification of PCa patients; (**H**) heatmap showing relationship of SRLPI score with common senescence markers; (**I**) Kaplan-Meier curve showing the survival difference of high- and low- SRLPI groups for PCa patients in TCGA database; (**J**) Kaplan-Meier curve showing the BCR-free survival difference of high- and low- SRLPI groups for PCa patients in GSE116918; (**K**) Kaplan-Meier curve showing the metastasis-free survival difference of high- and low- SRLPI groups for PCa patients in GSE116918; (**L**) ROC curve showing the diagnostic accuracy of SRLPI score for radiotherapy sensitivity in PCa patients. Abbreviations: SRLPI: senescence-related lncRNA prognostic index; BCR: biochemical recurrence; ROC: receiver operating characteristic curve; lncRNA: long non-coding RNA; PCa: prostate cancer. Note: prostate cancer patients were divided into high- and low- risk groups according to the median value of SRLPI score.

In the TCGA cohort, 47 lncRNAs were differently expressed between 498 tumor and 52 normal PCa samples (Figure 2A). 73 senescence-related lncRNAs and 38 BCR-related lncRNAs were found (Supplementary Figure 1). After intersection, we discovered 16 lncRNAs with differential expression linked to BCR and senescence (Figure 2B). Following multivariate Cox regression analysis, we utilized Lasso regression analysis to further screen lncRNAs, and when lambda equaled 0.02 (Figure 2C), we obtained three lncRNAs for the best model (Figure 2D), with small nucleolar RNA host gene 1 (SNHG1) and myocardial infarction associated transcript (MIAT) serving as the final independent risk lncRNAs (Figure 2E). Thus, we established a SRLPI score using the following formula: SRLPI = 0.700336146 × SNHG1 + 0.315877609 × MIAT. The correlation of outcomes and SRLPI score was presented in Figure 2F, and the diagnostic accuracy of SRLPI score for BCR was not good (Figure 2G). Notably, we found that SRLPI score was significantly associated with senescence markers (Figure 2H).

We divided the PCa patients into high- and low- risk groups according to the median value of SRLPI score. We found that high-SRLPI group was more susceptible to BCR than low-SRLPI group (HR: 3.33, *p* < 0.001; Figure 2I). Similar results were found in the GSE116918 [27] in terms of BCR-free survival (Figure 2J) and metastasis-free survival (Figure 2K). Surprisingly, SRLPI score showed highly diagnostic ability of radiosensitivity (AUC: 0.988; Figure 2L) using the GSE53902 [31]. In the multivariate Cox regression including clinical features, EAU and NCCN risk classifications and SRLPI score, this score was an independent risk factor in TCGA database (Supplementary Table 1) and GSE116918 [33] (Supplementary Table 2). In TCGA database, patients in high-SRLPI score had significantly higher age, Gleason score, T stage, N stage, positive lymphnodes and residual tumor (Table 1). Similar findings were observed in the GSE116918 [42] (Table 2). Furthermore, a substantial correlation between a higher SRPLPI score and older age was found in the GSE70768 [28] (Supplementary Figure 2).

**Table 1:** The clinical differences of the two risk groups in prostate cancer patients in TCGA database.

**Features**	**Low SRLPI score**	**High SRLPI score**	***P* value**
Sample	215	215	
Age, median (IQR)	61 (55, 65)	62 (57, 66.5)	0.016
Gleason score, *n* (%)			<0.001
6	19 (4.4%)	20 (4.7%)	
7	135 (31.4%)	71 (16.5%)	
8	20 (4.7%)	39 (9.1%)	
9	41 (9.5%)	85 (19.8%)	
T stage, *n* (%)			0.006
T2	93 (21.9%)	62 (14.6%)	
T3	116 (27.4%)	145 (34.2%)	
T4	3 (0.7%)	5 (1.2%)	
Race, *n* (%)			0.606
Asian	6 (1.4%)	5 (1.2%)	
Black or African American	22 (5.3%)	28 (6.7%)	
White	182 (43.8%)	173 (41.6%)	
*N* stage, *n* (%)			<0.001
N0	165 (44%)	141 (37.6%)	
N1	19 (5.1%)	50 (13.3%)	
Positive lymphnodes, *n* (%)			<0.001
No	160 (44.7%)	128 (35.8%)	
Yes	19 (5.3%)	51 (14.2%)	
Residual tumor, *n* (%)			<0.001
No	156 (37.2%)	117 (27.9%)	
Yes	52 (12.4%)	94 (22.4%)	

Abbreviations: IQR: interquartile range; SRLPI: senescence-related lncRNA prognostic index. Note: prostate cancer patients were divided into high- and low- risk groups according to the median value of SRLPI score.

**Table 2:** The clinical differences of the two risk groups in prostate cancer patients in the GSE116918.

**Features**	**Low SRLPI score**	**High SRLPI score**	***P* value**
Sample	124	124	
Age, median (IQR)	67 (64, 71.25)	69 (62, 73)	0.336
T stage, *n* (%)			0.002
T1	37 (16.6%)	14 (6.3%)	
T2	36 (16.1%)	40 (17.9%)	
T3	39 (17.5%)	53 (23.8%)	
T4	1 (0.4%)	3 (1.3%)	
Gleason score, *n* (%)			0.002
6	28 (11.3%)	14 (5.6%)	
7	52 (21%)	47 (19%)	
8	28 (11.3%)	24 (9.7%)	
9	16 (6.5%)	39 (15.7%)	

Abbreviations: IQR: interquartile range; SRLPI: senescence-related lncRNA prognostic index. Note: prostate cancer patients were divided into high- and low- risk groups according to the median value of SRLPI score.

### Mutation genes, functional enrichment, tumor heterogeneity and stemness

**Figure 3:**
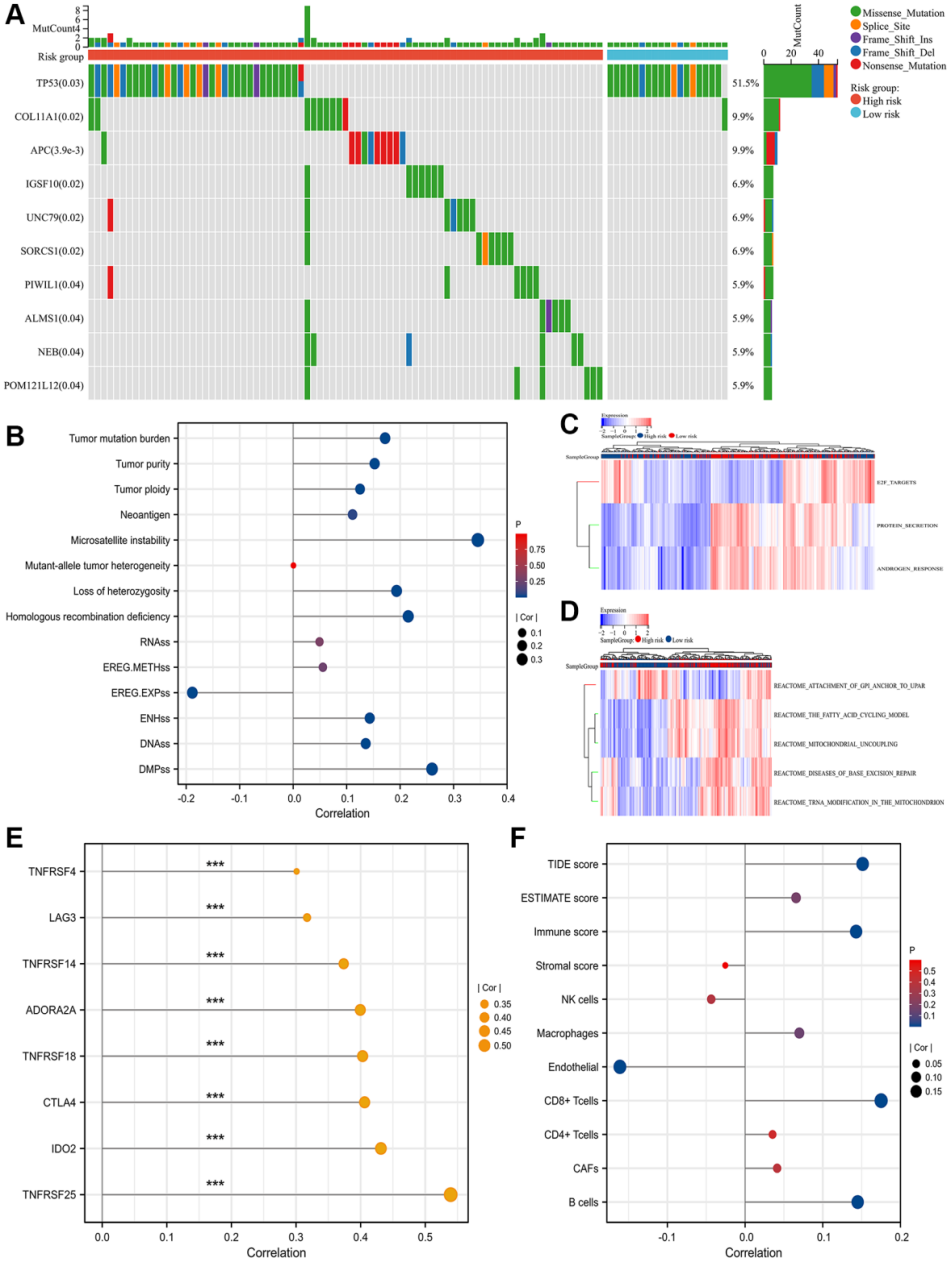
**Mutation genes, functional enrichment, tumor heterogeneity and stemness and TME.** (**A**) waterfall plot showing top 10 differentially mutation genes between high- and low- SRLPI groups for prostate cancer patients in TCGA database; (**B**) lollipop plot showing the relationship between tumor stemness and heterogeneity indicators and SRLPI score in TCGA database; (**C**) heatmap showing differences of enriched hallmarks between high- and low- SRLPI groups in TCGA database; (**D**) heatmap showing differences of enriched pathways between high- and low- SRLPI groups in TCGA database; (**E**) lollipop plot showing the relationship between significant immune checkpoints and SRLPI score with correlation coefficient ≥0.3 in TCGA database; (**F**) lollipop plot showing the relationship between TME scores and SRLPI score in TCGA database. Abbreviations: SRLPI: senescence-related lncRNA prognostic index; TME: tumor immune microenvironment; TIDE: tumor immune dysfunction and exclusion; CAFs: cancer-associated fibroblasts; NK: nature killer. Note: prostate cancer patients were divided into high- and low- risk groups according to the median value of SRLPI score.

The top gene between high- and low- SRLPI groups was tumor protein P53 (TP53) with statistical significance (Figure 3A). For tumor heterogeneity and stemness, SRLPI score was positively related to MSI, HRD, LOH, TMB, tumor purity, tumor ploidy, DMPss, ENHss and DNAss, but was negatively associated with EREG.EXPss with statistical significance (Figure 3B). Protein secretion and androgen response were found to be substantially enriched for the signature gene set enrichment in the low-SRLPI group, whereas E2F targets were found to be strongly enriched in the high-SRLPI group (Figure 3C). For pathway analysis, the fatty acid cycling model, mitochondrial uncoupling, disorders of base excision repair, and tRNA modification in the mitochondrion were substantially enriched in the high-SRLPI group, whereas attachment of glucose-6-phosphate isomerase anchor to urokinase plasminogen activator surface receptor was strongly enriched in the low-SRLPI group (Figure 3D).

### Tumor immune microenvironment (TME) and immune checkpoints

Immune checkpoints showed a substantial association between SRLPI score and indoleamine 2,3-Dioxygenase 2 (IDO2) and tumor necrosis factor receptor superfamily member 25 (TNFRSF25) levels with a value greater than 0.4 (Figure 3E). In the TME assessment, endothelium level had a statistically significant negative correlation with SRLPI score while B cells, CD8+ T cells, immune score, and TIDE score had a positive correlation (Figure 3F).

## DISCUSSION

The prevalence of malignant tumors in the elderly population has recently increased. PCa, a complex and heterogeneous population of molecular changes caused by heritable variants and epigenetic alterations, is difficult to diagnose and treat, despite the fact that the majority of patients are organ-confined [43, 44]. Fortunately, over the past ten years, research has advanced significantly. Several mutations with a significant PCa risk, such as breast cancer type 2 susceptibility protein (BRCA2) and homeobox B13 (HOXB13), were found [45, 46]. Additionally, it has been demonstrated that the expression of certain proteins is related to the response of androgen deprivation therapy (ADT) treatment in some cancer tissues, including androgen receptor (AR)-V7 [47]. One of the most often utilized therapies for PCa is radiation therapy; however, the tumor cells’ innate radioresistance limits local control and ultimately results in poor patient outcomes like recurrence, metastasis, and death. It is known that miRNAs may play a role in radioresistance, despite the fact that the underlying mechanisms are still not completely understood [48–50]. In contrast to miRNAs, lncRNAs can fold into secondary and tertiary structures and act as intended in a number of malignancies, including PCa [51, 52].

An increasing number of studies showing links between PCa and aging [1, 8, 14, 15, 32, 53]. Senescent cells can also inhibit carcinogenesis, promote tumor growth, recurrence, and PCa metastasis in advanced stages [54, 55]. Senescent tumor cells, on the other hand, can actually re-enter the cell cycle and acquire stem-like characteristics, which may indicate the possibility of recurrence [56–58]. In this study, from the perspective of cellular senescence, we preliminarily identified two senescence-related lncRNAs (SNGH1 and MIAT) and established a SRLPI score, both of which were significantly associated with BCR risk of PCa patients. SNHG1 is one of the most important regulatory RNAs in human cancer, acting as a competing endogenous RNA [59]. It was found to have enhanced expression in PCa and was associated with the proliferation, invasion, apoptosis and epithelial-mesenchymal transition (EMT) abilities of PC cell lines [60]. Meanwhile, MIAT has recently been found to be associated with the malignancy of PCa [61]. In this study, we again demonstrated their important role in PCa. Besides, SRLPI score was an independent risk factor of PCa patients, showing better advantages over EAU and NCCN risk classifications. We also suggested that further research is needed to determine whether these two lncRNAs contribute to the progression of PCa via senescence-related pathways. Interestingly, SRLPI score was positively related age in TCGA cohort and GSE70768 [28] rather than GSE116918 [27] and this score was significantly associated with senescence markers [62, 63]. These findings confirmed the concept that aging is not exactly the same as cellular senescence. With the advent of next-generation sequencing, TCGA project reported comprehensive molecular alterations of cancer patients and found that numerous genomic aberrations [64]. In this study, targeting mutations like TP53, COL11A1, APC and IGSF in high-SRLPI group might improve prognosis of such patients. Furthermore, we discovered that the SRLPI score showed excellent diagnostic capability of separating BCR for PCa patients.

Approximately 10–45% of PCa are radiation resistant, despite the fact that radiotherapy has a significant deal of effectiveness in treating advanced PCa [65]. The mechanism of how PCa resists radiation is not fully understood. *In vitro* experiments suggest that radiation-exposed PCa cell lines continued to produce adherent senescent-like cells that expressed common senescence-associated markers and non-adherent anoikis-resistant stem cell-like cells [66]. These surviving cells also displayed improved migration, increased androgen and epidermal growth factor receptor levels, and activation of their downstream Ras-MAPK, PI3K-Akt, and Jak-STAT pathways [67]. After radiation therapy, therapeutic failure for PCa is linked to testosterone production, cell proliferation, and apoptosis [68]. Base excision repair (BER) is the first-line DNA repair system in charge of maintaining genomic integrity to fend off illness, including cancer, and has the potential to influence tumor chemo- and radioresistance [69]. The pathway analysis revealed that BER-related diseases were more prevalent in the high-risk group. We hypothesize that targeting some DNA repair mechanisms, particularly BER, may be responsible for the rise in radiation-sensitive strategies in PCa. BER may be a significant driving force for PCa radioresistance [70]. In the high-SRLPI group, however, mitochondrial uncoupling is greatly enriched. We hypothesize that one of the causes of radioresistance in the high-risk group may be due to mild mitochondrial uncoupling, which mitigates oxidative stress and mitochondrial damage and protects cells from radiation-induced death [71].

AR is a ligand-activated transcription factor essential for both normal prostate development and tumorigenesis [72]. The formation and progression of localized and advanced metastatic PCa are caused by AR and its downstream signaling, which is the main oncogenic pathway in PCa [73]. It is well established that lncRNAs control androgen signaling through a variety of ways, including transactivating AR by interacting with their enhancer regions [74]. SNHG1 was directly found to be an androgen-responsive lncRNA with AR elements [75]. According to this study, the low SRLPI group was less responsive to BCR than the high SRLPI group. In earlier research, it was also found that high levels of SNHG1 were associated with a much-reduced rate of BCR and a shorter BCR-free survival [75]. Our previous study suggested that immune evasion may be a potential mechanism of BCR in PCa patients with similar results of immune checkpoints [10, 76]. It has been speculated that SNHG1 expression may be inhibited by AR activity in “androgen-dependent” tumors [75]. In terms of MIAT, Crea et al. identify it as a neuroendocrine PCa-specific lncRNA that is insensitive to all forms of hormone therapy [77]. We found that androgen responsiveness was highly enriched in the low SRLPI group, further suggesting that lncRNAs are part of the transition of PCa from hormone sensitive to castration resistant PCa. Additionally, we discovered that the high SRLPI group had a significant enrichment of E2F targets. It was discovered that the E2F transcription factor and AR interacted without the need for a ligand, and that androgen treatment changed the way that E2F1 bound to the Cdc6 promoter in PCa cells [78]. E2F1 expression in LNCaP prostate cancer cells deregulates androgen-dependent proliferation, reduces differentiation, and boosts death, according to *in vitro* tests by Libertini et al. [78]. In LNCaP cells, testosterone reciprocally regulates E2F activity in a biphasic manner [79]. We hypothesize that in BCR and PCa patients, the SRLPI score may control androgen translation through the E2F target, and that the E2F target is in turn controlled by androgen.

TMB and MSI were shown to be associated with the response to PD-1 treatments in solid tumors, such as bladder cancer and metastatic colorectal cancer [80, 81]. In this study, we found that patients with high SLRPI had higher TMB/MIS, and those with greater TMB/MSI had a higher propensity for BCR than their less-serious counterparts. We predicted that higher TMB/MSI patients could have worse ICB efficacy for PCa patients when combined with the correlation with TIDE score and results of TME assessment. In the immune microenvironment, we found that B cells, CD8+ T cells and immune scores were positively correlated with SRLPI scores, predicting poor immunotherapy efficacy in the high-risk group. These CD8+ T cells may be induced by genetic alterations associated with cancer [82]. Petitprez et al. also confirmed that clinical progression in PCa patients with positive lymph node nodules is correlated with CD8 + T cell infiltration. [83]. In addition, Guan et al. found that by boosting IFN expression, reduction of AR activity in CD8+ T cells reduced T cell fatigue and enhanced response to PD-1-targeted treatment. Targeting CD8+ T cells and lncRNA may help to overcome PCa immune resistance and poor prognosis given that increased CD8+ T cells were discovered to be associated with poor prognosis in this study [84]. Higher amounts of B cells were discovered in the high-risk group because recruitment of the chemokine CXCL13 to B cells in PCa promotes the development of castration-resistant prostate cancer by generating lymphotoxin [82]. Samples from PCa high-risk individuals and those with recurrence or progression of the disease displayed higher and more intratumoral CD20+ B-cell positivity [85].

The importance of lncRNAs in PCa prognosis prediction and treatment choice is being increasingly supported by research. The findings of the investigation demonstrate that the SRLPI score made up of lncRNA indicates a subset of high immune cells, but it is not good for the impact of immunotherapy and radiotherapy. BER and mitochondrial uncoupling are linked to this resistance. The control of androgen signaling, which may be partially mediated by E2F targets, is a key component of promising treatment approaches for lncRNAs. To the best of the author’s knowledge, our study is the first to use the TCGA database and GEO datasets to screen and validate the senescence-related BCR-determining lncRNA. In addition to being connected with BCR, the SRLPI score we created was also correlated with the primary PCa treatment modalities, including radiation, ADT, and immunotherapy, suggesting that lncRNAs are viable therapeutic targets.

## CONCLUSIONS

In this study, we identified and confirmed a prognosis- and radiosensitivity-related SRLPI score which might be useful in the clinical practice.

## Supplementary Materials

Supplementary Figures

Supplementary Tables
