# Supplementary material for: Identification of senescence-related lncRNA prognostic index correlating with prognosis and radiosensitivity in prostate cancer patients
Source: Aging (Albany NY). 2023 Sep 23;15(18):9358–76. doi: 10.18632/aging.204888 (PMC10564441; doi:10.18632/aging.204888)
Supplement: Supplementary Figures [file aging-15-204888-s001.pdf]

SUPPLEMENTARY FIGURES

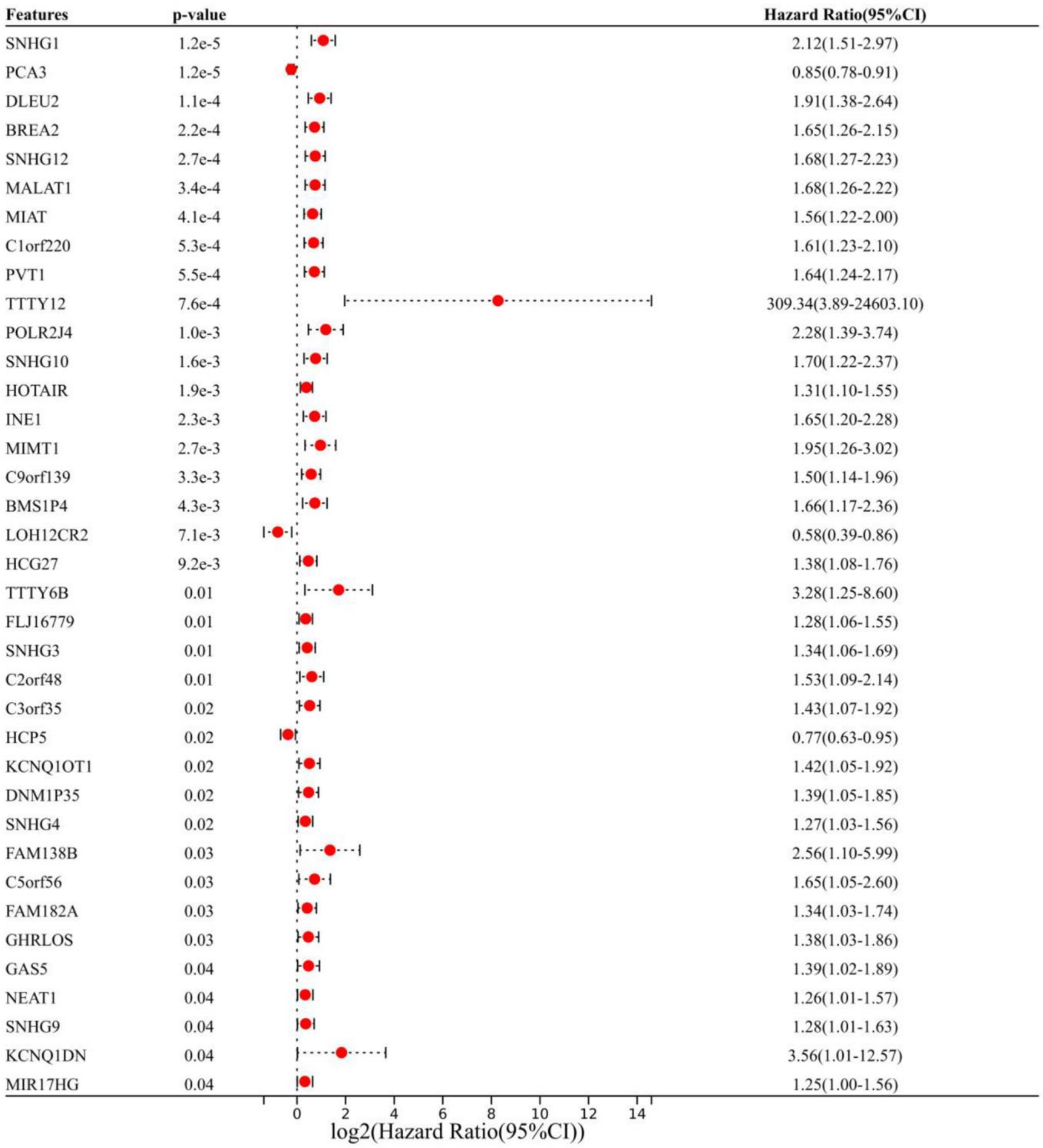

Supplementary Figure 1. The prognosis analysis of lncRNAs in TCGA database.

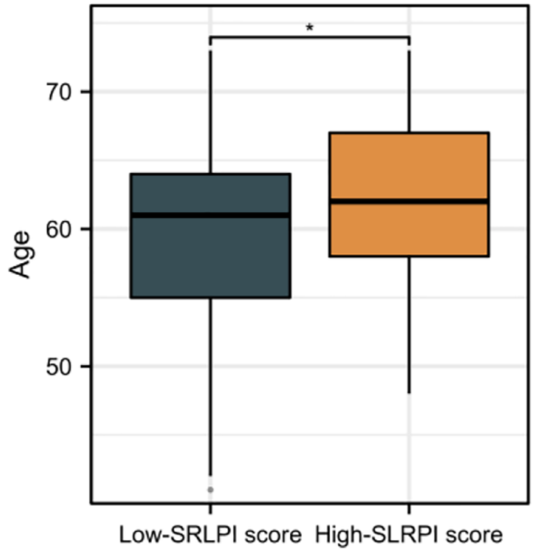

Supplementary Figure 2. The relationship of SRLPI score with age in the GSE70768.
