# Supplementary material for: Identification of senescence-related lncRNA prognostic index correlating with prognosis and radiosensitivity in prostate cancer patients
Source: Aging (Albany NY). 2023 Sep 23;15(18):9358–76. doi: 10.18632/aging.204888 (PMC10564441; doi:10.18632/aging.204888)
Supplement: Supplementary Tables [file aging-15-204888-s002.pdf]

SUPPLEMENTARY TABLES

Supplementary Table 1. Cox regression analysis results enrolling SRLPI score and clinical features in TCGA database.

Characteristics	Total (*N*)	Univariate analysis		Multivariate analysis	
		Hazard ratio (95% CI)	*P* value	Hazard ratio (95% CI)	*P* value
Risk group	430				
Low risk	215	Reference			
High risk	215	3.895 (2.125–7.136)	**<0.001**	3.479 (1.658–7.300)	**<0.001**
Age	430	1.016 (0.978–1.055)	0.426		
Gleason score	430				
GS6	39	Reference			
GS9	126	4.833 (1.157–20.194)	**0.031**	25546414.032 (0.000–Inf)	0.996
GS8	59	3.763 (0.832–17.011)	0.085	29899662.291 (0.000–Inf)	0.996
GS7	206	1.072 (0.242–4.756)	0.927	13611512.897 (0.000–Inf)	0.996
T stage	424				
T2	155	Reference			
T3	261	5.208 (2.230–12.163)	**<0.001**	3.494 (1.188–10.274)	**0.023**
T4	8	6.140 (1.235–30.532)	**0.027**	2.623 (0.266–25.817)	0.409
Race	416				
White	355	Reference			
Asian	11	0.673 (0.147–3.079)	0.610		
Black or African American	50	0.648 (0.288–1.460)	0.296		
N stage	375				
N0	306	Reference			
N1	69	1.822 (1.001–3.313)	**0.049**	84683287.020 (0.000–Inf)	0.999
Positive lymphnodes	358				
No	288	Reference			
Yes	70	1.937 (1.051–3.567)	**0.034**	0.000 (0.000–Inf)	0.999
Residual tumor	419				
No	273	Reference			
Yes	146	1.781 (1.050–3.019)	**0.032**	1.005 (0.542–1.865)	0.987
NCCN risk stratifications	427				
Low risk	27	Reference			
Intermediate risk	103	0.257 (0.036–1.827)	**0.174**	0.215 (0.000–Inf)	1.000
High risk	297	2.599 (0.633–10.678)	**0.185**	0.323 (0.000–Inf)	1.000
EAU risk stratifications	430				
Intermediate risk	133	Reference		Reference	
High risk	297	6.527 (2.362–18.036)	**<0.001**	1.497 (0.180–12.474)	0.709

Supplementary Table 2. Cox regression analysis results enrolling SRLPI score and clinical features in the GSE116918.

Characteristics	Total (*N*)	Univariate analysis		Multivariate analysis	
		Hazard ratio (95% CI)	*P* value	Hazard ratio (95% CI)	*P* value
Risk group	248				
Low risk	124	Reference			
High risk	124	1.832 (1.066–3.148)	**0.028**	1.957 (1.075–3.562)	**0.028**
Age	248	0.976 (0.937–1.017)	0.252		
T stage	223				
T2	76	Reference			
T3	92	1.728 (0.909–3.283)	0.095	1.747 (0.919–3.320)	0.089
T1	51	0.665 (0.255–1.732)	0.404	0.795 (0.301–2.098)	0.643
T4	4	7.971 (2.256–28.168)	**0.001**	6.475 (1.818–23.062)	**0.004**
Gleason score	248				
GS7	99	Reference			
GS8	52	1.083 (0.545–2.150)	0.821		
GS6	42	0.533 (0.216–1.316)	0.173		
GS9	55	1.310 (0.679–2.528)	0.421		
EAU risk stratifications	248				
Low risk	7	Reference		Reference	
Intermediate risk	68	0.380 (0.081–1.791)	0.221	0.343 (0.069–1.704)	0.191
High risk	173	0.971 (0.235–4.007)	0.968	0.797 (0.177–3.588)	0.767
NCCN risk stratifications	248				
Low risk	7	Reference		Reference	
Intermediate risk	68	0.380 (0.081–1.791)	0.221	0.343 (0.069–1.704)	0.191
High risk	173	0.971 (0.235–4.007)	0.968	0.797 (0.177–3.588)	0.767
